# Prevalence of *Listeria monocytogenes* in raw milk in Kerman, Iran 

**Published:** 2015-09-15

**Authors:** Ladan Mansouri-Najand, Mehrnoush Kianpour, Masoud Sami, Maziar Jajarmi

**Affiliations:** 1 *Department of Food Hygiene and Public Health, Faculty of Veterinary Medicine, Shahid Bahonar University of Kerman, Kerman, Iran;*; 2 *Graduate Student, Faculty of Veterinary Medicine, Shahid Bahonar University of Kerman, Kerman, Iran; *; 3 *Department of Pathobiology, Faculty of Veterinary Medicine, Shahid Bahonar University of Kerman, Kerman, Iran.*

**Keywords:** Food safety, Kerman, *Listeria monocytogenes*, Raw milk, Iran

## Abstract

*Listeria monocytogenes* as one of the most important pathogen in public health concerns is transmitted through consumption of contaminated food. The pathogen has been considered as a potential source of contamination of raw milk and dairy products. This research was aimed to investigate prevalence of *L. monocytogenes* in raw milk in Kerman region. In the summer of 2011, a total number of one hundred raw milk samples were collected from bulk tanks of some dairy farms and tested for *iap* and *actA *genes using polymerase chain reaction. Among the 100 samples, five isolates (5.0%) were detected as *L. monocytogenes* based on phenotypic and genotypic characteristics. Considering the low frequency of *L. monocytogenes* in this study, raw milk cannot be omitted as a potential source of food contamination for the population of the region. To achieve more accurate isolation, identification and control of *L. monocytogenes *in raw milk, it is suggested that new standard laboratory methods be implemented as well as biosafety outreach programs, management techniques and education.

## Introduction

*Listeria monocytogenes* is one of the most important pathogens in public health concerns.^1^ This type of pathogen is transmitted through food which is particularly challenging for people with immune problems.^2^^,^^3^ Listeriosis (*Listeria *infection) is relatively rare but statistics show that mortality rates can be high (up to 30.0%).^4^ The major means of transmission of *L. monocytogenes* to humans and animals is through consumption of contaminated food.^5^ The pathogen can be transmitted through various foods such as meat and meat products, fish and fish products, milk and dairy products (raw milk, soft cheeses etc.), vegetables and minimally processed food.^1^^,^^5^

There have been attempts to regulate amounts of* L. monocytogenes *in foods. In the USA, there should not be a bacterium in each 25 g of food.^6^ In Europe, this agent must not be above 100 CFU g^-1^ during the shelf life of food and there should be no *L.* *monocytogenes *in products that bacterium may grow in them.^7^

*Listeria monocytogenes* can grow and remain in raw milk in a broad range of temperatures, low pH and high salt concentration.^8^^,^^9^ Therefore, the pathogen has been found in sheep, goat and cow milk and is considered as a potential source of contamination of raw milk and dairy products, and outbreaks subsequently.^10^^,^^11^ Incidence of the pathogen has been reported at different rates (3.4% to 6.0%) in raw milk that can be contaminated from infected feces and milk.^12^^,^^13^

There are various methods for rapid detection of *L. monocytogenes, *one of which is polymerase chain reaction (PCR). It detects virulent genes in a food sample. Therefore, a number of important virulent genes are *plcB* (encoding phospholipase C), *hly* (listeriolysin O), *prfA* (transcriptional regulatory protein), *mpl* (metalloprotease), *actA* (Actin assembly-inducing protein A) and *iap* (Invasion associated protein).^14^^,^^15^

The region of Kerman in southeastern Iran, with a population of more than 700,000 is the case study used to test the prevalence of pathogens that present a concern to public health. In this city, there are several big and small suppliers of milk and milk products. This research was aimed to investigate prevalence of *L. monocytogenes* in raw milk in this region. In previous years *Listeria* had been considered as an important foodborne pathogen to threaten public health by many related organizations such as International Dairy Federation (IDF). These tests on milk samples in Kerman province are the first under Iran’s membership of the IDF. 

## Materials and Methods

Tests were done in the summer of 2011. A total number of one hundred raw milk samples were collected in sterile tubes from bulk tanks of some dairy farms in Kerman province. Once samples had been collected they were immediately transferred to the laboratory in conditions of 4 ˚C.

For each 25 mL sample of milk, 225 mL *Listeria *enrichment broth (Merck, Darmstadt, Germany) was added and then incubated at 30 ˚C. After 48 hr, 0.1 mL of enriched broth was cultivated on PALCAM *Listeria* selective agar medium (Merck) containing special supplements, at 37 ˚C for 48 hr. *Listeria* colonies were detected and confirmed by Gram staining, catalase production, oxidase activity and mobility tests. For identification of species, Microgen^TM^ Listeria-ID System (Microgen Bioproducts Ltd., Camberley, UK) was used according to the instructions. So *Listeria* isolates were evaluated in asculin hydrolysis, hemolysis, mannitol, xylose, arabitol, ribose, rhamnose, trehalose, tagatose, glucose-1-phosphate, methyl-D-glucose and methyl-D-mannose fermentation to identify species: *L. monocytogenes*, *L. ivanovii,* *L. seeligeri, L. inocua*, *L. welshimeri and L. grayi*. 

Also, artificially contaminated milk samples were prepared in serial dilution of bacterium. An overnight cultivation of *L. monocytogenes* ATCC 7644 was prepared in brain-heart infusion broth (BHI; Merck). The bacterium was diluted in raw milk (10^-1^ to 10^-15^) and finally, 0.1 mL of each dilution was cultured in three plates of PALCAM agar (Merck) for 24 hr at 37 ˚C.

DNA extraction was done by the boiling method, 3 to 4 pure colonies were suspended in 1 mL distilled water and centrifuged at 14000 rpm for 1 min. Supernatants were eliminated and the remains was washed using by 500 µL NaCl 0.85%. Tubes were boiled at 100 ˚C for 10 min and then immediately placed on ice. Strains were subjected to detecting *iap* and *actA* genes for duplex-PCR using primers described previously (Table 1).^16^^,^^17^ Total volume of PCR amplification reaction was 25 μL containing: 10 μLDNA template, 0.2 mM of each primer, 0.2 mM of each dNTP (Cinnagene, Tehran, Iran), 2.5 mM MgCl_2_ (Cinnagene), 2.5 μL 10x PCR buffer (Cinnagene), 1 unit *Taq* DNA polymerase(Cinnagene) and distilled water up to reaction volume. Also, the thermal program was 95 ˚C for 5 min (Initial denaturation), 95 ˚C for 1.5 min (Denaturation), 46 ˚C for 80 sec (Annealing), and 72 ˚C for 2 min (Elongation) that 35 cycles were repeated from the second step. Finally, terminal extension was performed at 72 ˚C for 7 min. Electrophoresis was done with 5 μL PCR products on 1.5% agarose gel for 75 min at 100 V in tris borate EDTA (TBE; Cinnagene) buffer (90 mM trisbase, 90 mM boric acid, 20 mM EDTA, pH 8.0) containing 0.5 μg mL^-1 ^ethidium bromide to visualize the amplicons. The electrophoresed gel was analyzed by UV trans-illumination. *Listeria monocytogenes* ATCC 7644 strain and distilled water was used as the positive and no-template negative controls, respectively, and specific bands were compared with 50-bp DNA ladder (Vivantis, Selangor, Malaysia).

**Table 1:** Primers used in the PCR reactions

**Tareget**	**Primer**	**Sequence 5'-3'**	**Product size**	**Reference**
***actA***	actA-F	GTGATAAAATCGACGAAAATCC	400 or 300 bp	Vazquez-Boland *et al.* ^16^
	actA-R	CTTGTAAAACTAGAATCTAGCG		
***iap***	MAR 1	GGGCTTTATCCATAAAATA	453 bp	Manzano *et al.* ^17^
	MAR 2	TTGGAAGAACCTTGATTA		

## Results

Among the 100 samples, five isolates (5.0%) were detected as *L. monocytogenes* based on phenotypic characteristics. Those black with black sunken colonies in PALCAM *Listeria* selective agar mediums were Gram positive, catalase-positive and oxidase-negative. Also, these isolates possessed β hemolysis in sheep blood agar and tumbling motility at 25 ˚C and 37 ˚C. These strains were confirmed by Microgen^TM^ Listeria–ID System as* L. monocytogenes*: asculin hydrolysis, hemolysis, arabitol, rhamnose, trehalose, methyl-D-glucose and methyl-D-mannose fermentation was positive but mannitol, xylose, ribose, tagatose and glucose-1-phosphate fermentation was negative. All five *Listeria *strains were identified as *L. monocytogenes *and showed presence of *iap* and *actA* genes in PCR. In addition, five colonies were raised from 0.1 mL belonging to 10^-13^ dilution of bacterium in artificially contaminated milk samples. Thus, this method was determined as sensitive to 50 CFU mL^-1^.

## Discussion

This study determined that prevalence of *L. monocytogenes *in bulk tank, raw milk samples taken in the Kerman region, was low (5.0%). Results from tests in other areas in Iran have been reported as follows; 20% in Esfahan, 4.0% in Mashhad, 1.7% and 3.3% from two different dairy farms northern Iran.^18^^-^^20^ There have also been reports on prevalence of *L. monocytogenes *in milk samples from USA determined at 6.5%, Swedish dairy farm 1.0%, Morocco 5.9%, Latvia 1.4% and Syria with a considerably higher rate (41.6%).^21^^-^^25^

Results reported from the tests in this study are in agreement with reports on samples from Mashhad, east northern Iran and Latvia, while the rates of prevalence determined by tests in Esfahan, USA, Morocco and Syria were relatively higher and those in Sweden were lower.^18^^-^^20^ The variable rate of prevalence depends to some extent on hygiene, dairy management, the number of animals on a farm, season, geographical characteristics of a region, sampling technique and laboratory detection method.^19^^,^^26^ The most important risk factors of *L. monocytogenes* contamination of raw milk include defective disinfection of teats before milking, lack of correct management of barn and silage, insufficient hygiene practice in the environment and a low level of cleanliness among cows.^27^ Milking system and hygienic control of milking are considerations that have a considerable statistically relationship with risk of *Listeria *persistent in bulk tank milk.^28^ Therefore, the most important challenge during milking is exogenous contamination of milk with fecal material due to bad practice of hygiene standards.^29^ There are also challenges of good practice during transportation and storage of milk that need consideration.^30^^,^^31^

Although, prevalence of* L. monocytogenes* in this research was low, the importance of pasteurization and processing of dairy products (especially raw milk) should not be overlooked.^32^ Persons who are related to dairy farms such as producers and workers are at risk of oral infection by contaminated raw milk.^25^

In this study, all five isolates identified as *L. monocytogenes *were also positive for *iap *and *actA* genes. There is differences in the level of contamination with *Listeria *species between traditional foods.^10^ Proteins coded by *iap* gene are present in *Listeria *spp., however, they are different in each species and can be used for diagnostic targets by PCR.^33^ The *actA* gene is also appropriate for epidemiological approaches. This PCR-based assay can be considered as a specific method for recognize the DNA from different *L. monocytogenes* strains of raw milk, but it needs to more investigations.

In conclusion, considering the low frequency of *L. monocytogenes* in this study, raw milk cannot be omitted as a potential source of food contamination for the population of the region. It is recommended that a larger sample size should be employed selected from more dairy farms in order to make better and more precise judgment on pre-valence of *L. monocytogenes. *Thus, to achieve more accurate isolation, identification and control of *L. monocytogenes *in raw milk, it is suggested that using new standard laboratory methods be implemented as well as biosafety outreach programs, management techniques and education. 
